# Cryoablation therapy stimulates the antigen-specific T cell immune responses generated by therapeutic HPV DNA vaccine

**DOI:** 10.1186/2051-1426-3-S2-P364

**Published:** 2015-11-04

**Authors:** Sung Yong Lee, Kyung Hoon Min, Gyu Young Hur, Je Hyeong Kim, Jae Jeong Shim, Kyung Ho Kang, Kye Young Lee, Chien-Fu Hung, TC Wu

**Affiliations:** 1 Korea University Medical Center, Seoul, Republic Of Korea; 2 Korea University Medical Center, Seoul, South Korea; 3 Korea University Medical Center, Ansan, South Korea; 4 Konkuk University, School of Medicine, Seoul, South Korea; 5 Johns Hopkins Medical Institution, Baltimore, MD, USA

## 

Antigen-specific DNA vaccine immunotherapy is an attractive approach for the treatment of cancer since it has the potency to specifically eradicate systemic tumors without damaging the normal cells. However, DNA vaccine shows insufficient immune response for antigen presenting cells and has minimal clinical efficacies. Cryoablation therapy is the therapeutic application of extreme cold for localized destruction of living tissue. It can develop cancer cell necrosis and release damage-associated molecular patterns (DAMPs) in tumor microenvironment. According to other reports, cryotherapy has immunogenic potential. Therefore, we explored the employment of cryoablation in combination with HPV E7 specific CRT/E7 DNA vaccine in a preclinical mouse model. For *in vivo* experiment, the mice were divided into four groups: group 1 received no treatment after the E7-expressing TC-1 tumor challenge, group 2 was treated with cryoablation therapy, group 3 was immunized with the CRT/E7 DNA vaccine, and group 4 was both immunized and received cryoablation therapy. Mice were monitored for E7-specific CD8 (+) T cell immune responses, myeloid-derived suppressor cells (MDSCs) and antitumor effects. *In vitro* assay, TC-1 cells were incubated in deep freezing and thawed for determination of HMGB1 from the supernatant. The combination therapy of cryoablation and CRT/E7 DNA vaccination significantly inhibited tumor growth and increased E7-specific CD8+ T cells in spleen compared to other treatment groups. Furthermore, the immunosuppressive MDSCs in tumor mass were significantly decreased in combination therapy group. We found that treatment with deep freezing and thawing led to up-regulation of HMGB1 in TC-1 tumor cells, rendering the tumor cells more susceptible to APCs and E7-specific CD8+ T cell-mediated killing. In conclusion, cryoablation therapy can enhance therapeutic HPV DNA vaccine potency in generating improved antigen-specific immune responses and antitumor effects through the DAMP signal. These findings have important implications for future clinical translation.

